# Relationship between occupational noise exposure and the risk factors of cardiovascular disease in China: A meta-analysis

**DOI:** 10.1097/MD.0000000000011720

**Published:** 2018-07-27

**Authors:** Ye Yang, Enguo Zhang, Juan Zhang, Shangya Chen, Gongchang Yu, Xiaoshan Liu, Cheng Peng, Martin F. Lavin, Zhongjun Du, Hua Shao

**Affiliations:** a Department of Toxicology, Shandong Academy of Occupational Health and Occupational Medicine; b School of Medicine and Life Sciences, University of Jinan, Shandong Academy of Medical Sciences; c Department of Radiology, Shandong Tumor Hospital, Affiliated to Shandong Academy of Medical Sciences, Jinan, Shandong, People's Republic of China; d Queensland Alliance for Environmental Health Sciences, the University of Queensland, Brisbane, Queensland; e University of Queensland Centre for Clinical Research, the University of Queensland, Herston, Brisbane, Australia.

**Keywords:** blood pressure, electrocardiograph, meta-analysis, occupational noise, risk factors

## Abstract

**Background:**

At present, occupational noise exposure has become one of the risk factors of occupational workers and attracted serious concerned of most of occupational disease researchers. To assess associations of occupational noise exposure and cardiovascular disease by meta-analysis.

**Methods and analysis:**

Results from primary studies about occupational noise and cardiovascular disease (2000–2017) were retrieved from literatures, which were conducted in China only. Both random and fixed effect model were used to calculate pooled odds ratio (OR) and their corresponding 95% confidence interval (CI). Review Manager and Stata software were used to perform data analysis. This study followed the Preferred Reporting Items for Systematic Reviews and Meta-Analysis statements.

**Results:**

After applying stringent inclusion and exclusion criteria, 4771 exposures and 3068 controls from 11 primary studies were used to analyze the relationship between occupational noise exposure and cardiovascular disease. The risk of developing high blood pressure for workers exposed to noise is 2.55 times higher than the controls (*I*^*2*^ = 52%, 95% CI: 1.94–3.36), and electrocardiograph (ECG) abnormality is 2.27 times higher than the control groups (*I*^*2*^ = 22%, 95% CI: 1.96–2.62). The bias analysis suggested that there is publication bias, but it didn’t affect the conclusions from trim test.

**Conclusion:**

The impact of high-intensity noise exposure on the worker's cardiovascular system is much greater than that of the unexposed control group, and the effect on hypertension of the exposed group is greater than that of the ECG.

## 1 Introduction

According to WHO, there were more than 36 million people died of non-communicable diseases each year from all over the world. Non-communicable diseases include cardiovascular diseases, diabetes, respiratory diseases, and even cancer, accounting for 2/3 of the world's total deaths. At present, cardiovascular and cerebrovascular disease has become the first cause of death, accounting for more than 40% of the total number of deaths. “China's cardiovascular health index (2017)” proposed that there were more than 290 million people suffering from cardiovascular disease in China. Therefore, the study of cardiovascular disease and its risk factors are of great significance in clinical medicine and public health.

China is the largest industrial country with the largest industrial and labor force in the world. Professional workers, who may be exposed to the physical or chemical harmful substances during their work and are not effectively protected, may cause serious occupational diseases. This also shows that the current status of occupational diseases in China is not optimistic. Noise is a common physical and harmful factor in industrial processes, which can be found in different workplaces and can directly lead to the occurrence of occupational noise-induced deafness. Mild exposure can not only cause noise deafness and other hearing loss diseases, but also, in severe cases, can cause cardiovascular damage or even death. Cardiovascular diseases are closely related to the working environment.^[1]^ Exposure to occupational noise exposure or industrial noise during industrial induction has a negative impact on the health of workers. Continual exposure to high intensity noise would make people feel uncomfortable. The sound pressure level (SPL) of the fan is 30 dBA, the washing machine is more than 50 dBA, the air conditioner is about 70 dBA, the printing workshop reaches 90 dBA, and the noise of the aircraft is more than 115 dBA. Long exposure in the environment with SPL over 90 dBA will lead to serious damage in auditory system and cause diseases such as neurasthenia, headache, high blood pressure and others. Noise above the pain threshold (100 dBA), will cause ear swelling and pain. The noise level above 115 dBA could damage the function of the cerebral cortex. If the noise level is higher than 175 dBA, heart resonance could occur and may lead to death. The effect of noise on the cardiovascular system can be chronic and sustainable. Long-term low-frequency noise can cause sleep disorders, headaches, tinnitus, and other social and psychological problems, and death can occur in severe cases.

Noise is also suggested to have effects on various organs and systems such as gastrointestinal,^[2]^ respiratory,^[3]^ immune,^[4]^ reproductive,^[5]^ and neurogenic system.^[6]^ Among all, the impact of noise on the cardiovascular system is particularly prominent, according to several studies.^[7–9]^ As a possible risk factor for cardiovascular disease, noise can be extremely harmful by accelerating heart senescence and increases the incidence of myocardial infarction, and to a certain extent. However, in contrast to extensive study on its damaging effect on auditory system, noise's damage on non-auditory system has not attracted widespread attention.

In this study, we explored the effects of high intensity noise exposure on the cardiovascular system by performing systematic quantitative analysis of the published studies in China. In order to gain a better understanding of the relationship between noise exposure and cardiovascular disease in China, we conducted a meta-analysis of the relevant case-control studies and cohort studies to analyze the association of noise with cardiovascular disease. A large number of foreign researchers have come to the conclusion that the relationship between occupational noise exposure and cardiovascular disease, in other countries, has been confirmed.^[8]^ However, the dose response relationship between long-term exposure and heart functions and relevant indications or diagnosis criteria remain to be fully addressed.

Some of the findings suggested that noise is harmful to cardiovascular system. Noise has cumulative effects of causing irreversible damages on workers’ heart function.^[10]^ Studies have shown that cumulative noise exposure was a significant predictor of diastolic blood pressure, indicating that noise exposure has a direct or indirect impact to changes in blood pressure.^[11]^ The effect of noise on the cardiovascular system is related to the time and intensity of exposure to noise.^[12]^ A study using rat model indicated exposure to high-intensity noise for a long time can cause high blood pressure, and the performance problem.^[13]^

Electrocardiograph (ECG) can often be one of the important indicators for studying noise and its association with cardiovascular disease. This is because ECG alterations happen before hearing loss and can be used as noise indicators of early damage to workers.^[14]^ There is a dose-response relationship between cumulative noise exposure and abnormal ECG, which can be used as damage index.^[15]^ High blood pressure is another common indicator that can be used as a indicator of noise-induced cardiovascular disease inspection.^[16]^ According to the current study, long-term exposure to noise can increase the secretion of adrenal glands in the body, leading to the rise in blood pressure.^[17]^

By using the meta-analysis, we intended to understand the current status of cardiovascular disease in current exposed workers, and to assess the different variables in the diagnostic criteria such as blood pressure and ECG for cardiovascular disease and occupational noise, the actual survey data in the exposure group and the control group were combined to make a systematic evaluation.

## 2 Materials and methods

The meta-analysis of the preferred reporting items for systematic reviews and meta-analysis (PRISMA) guidelines were followed for the current study. The guideline contains a checklist of 27 projects, which provide the specific requirements of each project.^[18]^

We searched for research articles studying the occupational noise exposure and cardiovascular disease in China, which published in the domestic or international biomedical journals from 2000 to 2017. More than 400 articles in the language of Chinese and English were retrieved. The search was performed searching the following keywords cardiovascular AND occupational noise AND China.

We retrieve data from the following databases to obtain comprehensive information: Pubmed, Embase, Chinese Science Periodical Database, Chinese Science and Technology Journal Database, and Chinese Journal Full–text Database.

### 2.1 Inclusion criteria

According to the specified inclusion and exclusion criteria, we included and excluded studies by reading the title and abstract. The studies included in this meta-analysis were all from Chinese region and the research objects were Chinese workers by searching in the data base for Chinese literatures. All analyses in our study were based on previous published studies, thus no ethical approval and patient consent are required.

We used The Newcastle-Ottawa Scale (NOS) to assess the quality of the literatures from following aspects. The study design is a cross sectional study or a cohort study. The studies have sufficient sample data (the exposure group and the control group) with reliable odds ratio (OR) and 95% confidence interval. In line with the diagnostic criteria of cardiovascular disease. According to the 1999 World Health Organization/ International Hypertension Society treatment guidelines, high blood pressure (systolic blood pressure ≥ 140 mmHg, diastolic blood pressure ≥ 90 mmHg) and ECG abnormalities were used to analysis. Exposure factor is occupational noise (SPL > 85 dBA) and there are specific instructions for the detection of occupational noise exposure intensity. The inclusion of the design and research methods are similar and the data are complete.

### 2.2 Exclusion criteria

The studies that were not dealing with noise relevant for work-related exposure, as well as those in languages other than Chinese. Newly employed workers were excluded because of short working time. Workers who had the history or family history of cardiovascular disease, hypertension or cerebrovascular diseases that can be the confounding effect of the study.

### 2.3 Statistical analysis

We used RevMan 5. 2 software (Copenhage: The Nordic Cochrane Centre) for data analysis and report the pooled effect in OR. The heterogeneity was checked using Q test. If the *P* value is greater than .05 (and *I*^*2*^ less than 50%), it shows that there is no heterogeneity between multiple studies and the OR from fixed effect model is reported. If the *P* value is less than .05 (and *I*^*2*^ greater than 50%), it indicates that there is a heterogeneity, and the OR from random effect model is reported.

We use Stata 12.0 software (StataCorp., College Station, Texas) for sensitivity analysis, to assess the stability of the literature and the credibility of the results. Funnel and Egger test is applied to check whether there is a publication bias across the studies. If there is a publication bias among studies, we use the trim method to check if the effect is changed before and after the trimming to determine whether the results are stable.

## 3 Results

### 3.1 Characteristic of participants

Eleven studies with a total of 7839 samples (4771 exposures and 3068 controls) were included in this study. In this article, the main diagnostic indicators for cardiovascular disease are blood pressure measurement and ECG measurements. In Tables 1 and 2,^[19–29]^ we summarize the main characteristic of samples from each study.

**Table 1:**
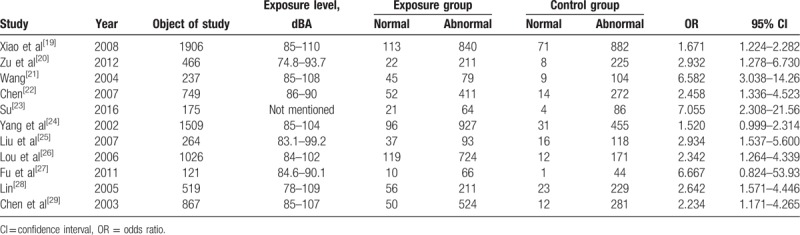
Included studies of hypertension in the meta-analysis.

**Table 2:**
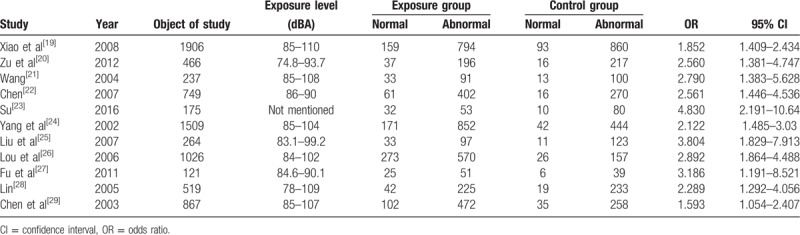
Included studies of electrocardiographic abnormalities in the meta-analysis.

### 3.2 Meta-analysis

**Figure 1:**
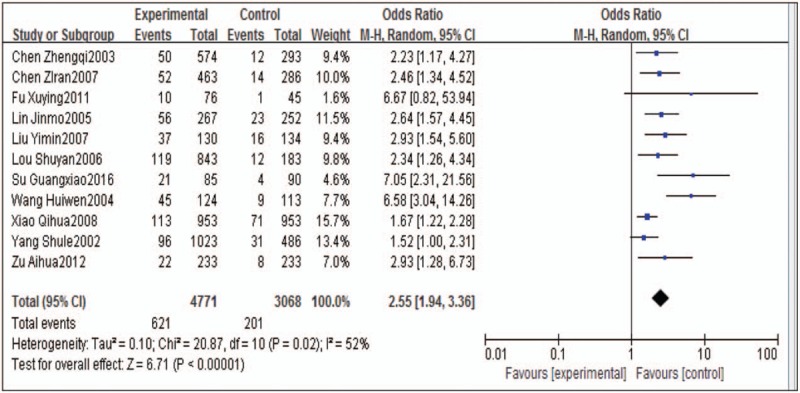
Forrest plot of the association between occupational noise exposure and blood pressure in meta-analysis. The random effects model was used to analyze the data of 11 articles. CI = confidence interval, OR = odds ratio.

The effects of occupational noise exposure on workers’ blood pressure from 11 studies were statistically combined with OR values. The results of heterogeneity test showed that Q = 20.87, *P* = .02 < .05, *I*^*2*^ = 52%, suggesting that there was moderate heterogeneity across multiple studies and hence we analyzed the data using random effect model. The results showed that the risk of cardiovascular disease for workers exposed to noise is 2.55 times higher than the controls (95% confidence interval, CI: 1.94–3.36), and association *P* < .01 (Z = 6.71), which is statistically significant (Fig. 1).

**Figure 2:**
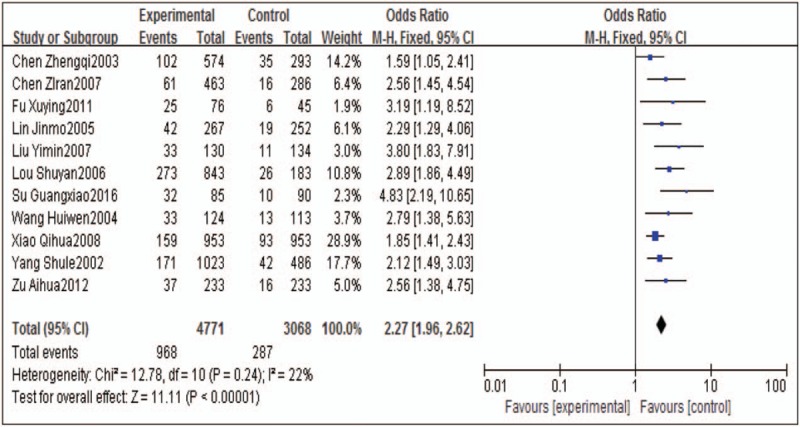
Forrest plot of the association between occupational noise exposure and ECG in meta-analysis. The fixed effects model was used to analyze the data of 11 articles. CI = confidence interval, ECG = electrocardiograph, OR = odds ratio.

Similarly, we also analyzed the abnormal ECG data of the workers. The heterogeneity test results indicated that Q = 12.78, *P* = .24 > .05, *I*^*2*^ = 22%, suggesting that the data for electrocardiogram abnormality have a little heterogeneity. Hence, results from fixed effect Model are reported. The results showed that the ECG abnormality of the workers exposed to occupational noise was 2.27 times higher than that of the control group (95% CI: 1.96–2.62), and the association is statistically significant (Z = 11.11, *P* < .01) (Fig. 2).

### 3.3 Sensitivity test

**Figure 3:**
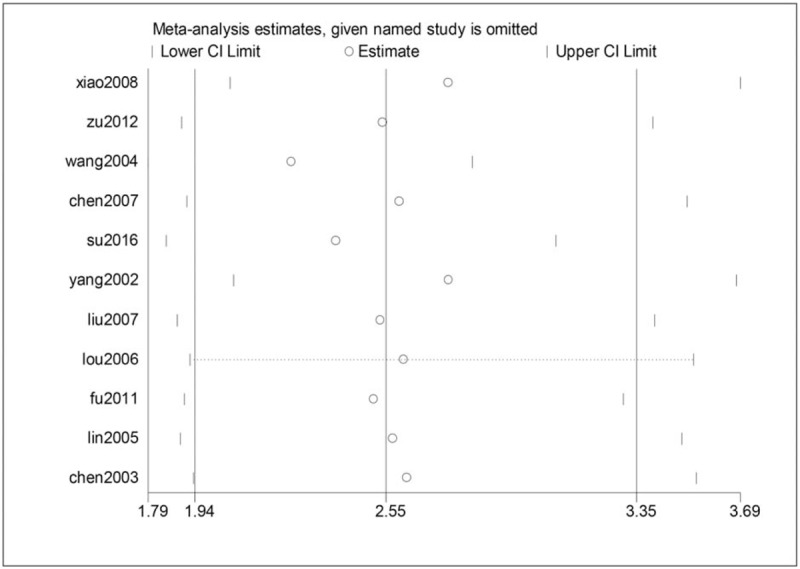
Sensitivity test of meta-analysis.

As the heterogeneity test in the meta-analysis of noise and blood pressure indicated the presence of moderate heterogeneity, we applied sensitivity test to analyze. The result suggests that Wang Huiwen's research has the greatest heterogeneity (Fig. 3). After excluding Wang Huiwen's data from our analysis, the heterogeneity score of the meta-analysis was reduced (*I*^*2*^ = 30). This indicates that results from the 10 studies are consistent.

### 3.4 Bias analysis

**Figure 4:**
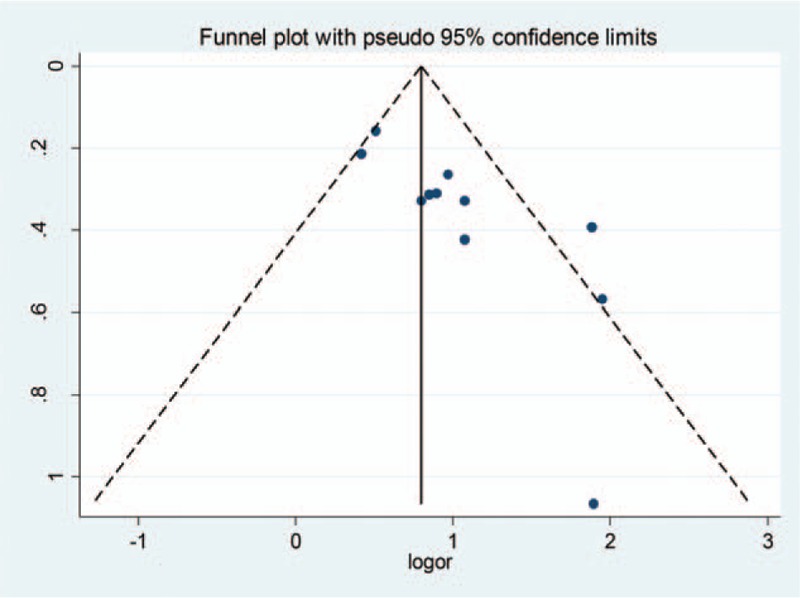
Funnel plot of meta-analysis (95% CI). CI = confidence interval.

Apparent asymmetry in the funnel plot indicates publication bias in the study (Fig. 4). We use trim method to observe the variation of the effect before and after the trimming to determine whether the results are stable. According to trim method's results, the results did not change significantly after addition of 5 studies, suggesting that the outcome was stable. The results showed that the Q value and *P* value of the heterogeneity test were both less than .05, suggesting that the results of bias after trimming were not affected by the meta-analysis results (Table 3).

**Table 3:**
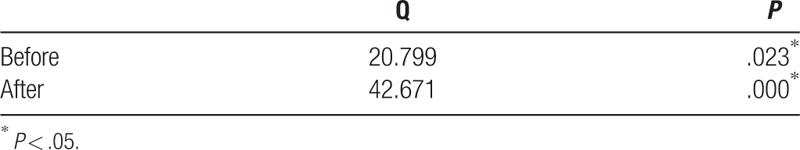
The results of the effect of the trim method.

## 4 Discussion

There have been reviews and studies that have confirmed that occupational noise has an positive^[30]^ with blood pressure. In this article, a meta-analysis based on cardiovascular and blood pressure and ECG abnormalities can provide a more direct and powerful demonstration of the positive correlation between noise and cardiovascular disease. A large amount of noise pollution poses a serious threat to the workers. Noise has a cumulative effect on hearing impairment, and the degree of hearing loss after prolonged exposure depends not only on the intensity of the noise, but also on the type of noise and the time of contact and the mode of contact.^[31]^According to the results of this meta-analysis, long term and high intensity (sound pressure level greater than 85 dBA) of exposure to occupational noise can lead to cardiovascular disease. Noise can make the vascular smooth muscle improved sensitivity and reactivity to contraction of vascular material stimulation, resulting in increased vascular contraction response led to increased blood pressure. Long-term exposure to noise can lead to vasoconstriction, rapid reduction in blood volume, and the greater the noise intensity, the greater the vasoconstriction. In the production life, workers exposed to noise for a long time, can cause damage to auditory system and other related systems. Blood pressure and electrocardiogram measurements have become the most common research indicators in the cardiovascular system because of their intuition and simplicity.

Meta-analysis is distinguished from the traditional systematic review by using more intuitive quantitative data to merge the results, increasing the confidence of the conclusions. This meta-analysis incorporated survey data from multiple studies and combined by statistical methods to improve the statistical performance of the original data.^[32]^ This meta-analysis concluded with a reliable conclusion that the impact of high-intensity noise exposure on the worker's cardiovascular system is much greater than that of the unexposed control group and the effect of hypertension and electrocardiogram on the exposed group are greater than that of the unexposed group. And all the literature included in this meta-analysis suggested that noise can cause an increase in blood pressure and electrocardiogram fluctuations.

Some studies have suggested that noise can produce a series of general stressful, especially cardiovascular reactions, because the noise is a stressor and it can cause changes in stress-related hormones.^[33]^ Several researchers have studied the relationship between occupational noise exposure or abnormalities in hypertension and electrocardiogram. Animals and human trials have confirmed that noise can cause fluctuations in blood pressure. However, some epidemiological studies have shown that portion of workers are adaptive to noise, and prolonged exposure do not produce discomfort, resulting in a negative outcome.^[34]^ There have been studies that shows high-intensity occupational noise exposure can speed up or slow heart rate, but in this meta-analysis, we found that prolonged high-intensity noise exposure can significantly increase the risk of hypertension and ECG abnormalities, and lead to cardiovascular disease. The prevalence of hypertension and ECG abnormalities in the exposed group was significantly higher than that in the control group. It was found that high-intensity noise exposure was one of the risk factors for cardiovascular disease.^[35]^

However, the evidence that the relationship between noise and cardiovascular disease is still not conclusive, not only because of the complexity of the noise impact on health problems, but also because of different exposure characteristics, other confounding factors, and publication bias.^[12]^ Changes in blood pressure and electrocardiogram are not the only diagnostic indicators of cardiovascular disease, so the relationship between cardiovascular disease and occupational noise due to other factors (myocardial infarction, myocardial ischemia, and so on) remains to be explored. Other studies have shown that the combination of noise and high temperature has a greater impact on the cardiovascular system than noise alone.^[36]^ So in further study of cardiovascular disease in occupational workers, the synergies should be considered in many ways.

## 5 Conclusions

In conclusion, the strongest evidence of a relationship between occupational noise and cardiovascular effects relates to studies of hypertension and ECG abnormalities. Noise in work gives an increasing effect on the cardiovascular disease. In the course of practice, we should strengthen the protection of staff in the noise environment, and we can wear protective gear or control noise to reduce noise on the human body damage.

## Acknowledgments

We thank the financial support from the National Natural Science Foundation of China (81602893), Natural Science Foundation of Shandong Province (ZR2015YL049), Medical and Health Technology Development Plan Project of Shandong Province (2016WS0540), Key Research and Development Plan of Shandong Province (2017GSF18186, 2018GSF118018), and Innovation Project of Shandong Academy of Medical Science.

## Author contributions

HS and ZJD obtained funding for the study. HS and ZJD designed the study, wrote and revised the article. YY and EGZ performed the analysis and interpretation of data, and drafted the article. JZ, SYC, and GCY performed the analysis and interpretation of data. XSL, CP, and MFL provided technical support for the analysis and critical revision of the article. All authors read and approved the final article.

**Conceptualization:** Zhongjun Du.

**Data curation:** Ye Yang, Enguo Zhang, Juan Zhang, Shangya Chen, Xiaoshan Liu.

**Formal analysis:** Shangya Chen, Hua Shao.

**Funding acquisition:** Zhongjun Du, Hua Shao.

**Investigation:** Gongchang Yu.

**Methodology:** Gongchang Yu, Xiaoshan Liu, Cheng Peng, Martin Lavin, Hua Shao.

**Project administration:** Zhongjun Du.

**Supervision:** Zhongjun Du.

**Writing – original draft:** Ye Yang, Enguo Zhang.

**Writing – review & editing:** Cheng Peng, Martin Lavin, Zhongjun Du, Hua Shao.
